# Extracorporeal Multiorgan Support During Prone Positioning in Severe ARDS: A Physiologic Proof-of-Concept Study

**DOI:** 10.3390/life16091578

**Published:** 2026-09-21

**Authors:** Tobias Lahmer, Eva Ortner, Aritz Perez Ruiz de Garibay, Luigi Camporota, Bernd Panholzer, Roland M. Schmid, Johanna Erber, Fabian Kleinhenz, Luis Hinterwaldner, Lailai Qu, Julian Triebelhorn, Miriam Dibos, Ulrich Mayr

**Affiliations:** 1 TUM School of Medicine and Health, Clinical Department of Internal Medicine II, TUM University Hospital, Ismaninger Str. 22, 81675 Munich, Germany; 2 Idazmed Consulting SL, 01006 Vitoria-Gasteiz, Spain; 3 Division of Anaesthetics, Pain Medicine and Intensive Care (APMIC), Department of Surgery and Cancer, Faculty of Medicine, Imperial College London, London W12 0NN, UK; 4 Department for Cardiac Surgery, University Hospital Schleswig-Holstein, 24105 Kiel, Germany

**Keywords:** acute respiratory distress syndrome, prone positioning, extracorporeal carbon dioxide removal, advanced organ support, hypercapnia, mechanical ventilation

## Abstract

Background: Prone positioning and lung-protective ventilation are key components of the management of moderate-to-severe acute respiratory distress syndrome (ARDS). However, protective ventilation may be limited by refractory hypercapnia, acidosis, and hemodynamic instability. Whether extracorporeal multiorgan support can complement prone positioning by alleviating these physiologic constraints remains unknown. Methods: We conducted a retrospective physiologic cohort study of adult patients with severe ARDS treated with prone positioning combined with extracorporeal multiorgan support using the ADVOS system. The cohort represented a highly selected population with bacterial pneumonia-associated ARDS, sepsis, acute kidney injury, and vasopressor dependence. Physiologic variables were recorded from baseline to the end of the first (t16) and second (t40) prone sessions. The primary objective was to assess short-term changes in arterial pH, PaCO_2_, PaO_2_/FiO_2_ ratio, driving pressure, and respiratory system compliance. Results: Fifteen patients were included. Median baseline PaCO_2_ was 68.1 mmHg and pH was 7.22. By t16, PaCO_2_ decreased to 42.6 mmHg (*p* = 0.001) and pH increased to 7.41 (*p* = 0.001). The PaO_2_/FiO_2_ ratio increased from 82 to 146 (*p* = 0.001), and driving pressure decreased from 20 to 17 cmH_2_O (*p* = 0.003). Mechanical power decreased from 28.5 to 25.9 J/min (*p* = 0.033), whereas respiratory rate and respiratory system compliance did not reach statistical significance at t16. Vasopressor requirements also decreased during the observation period. Extracorporeal support was maintained throughout prone positioning in all patients. Conclusions: In this highly selected cohort, combined prone positioning and extracorporeal multiorgan support was associated with rapid correction of hypercapnic acidosis, improved oxygenation, and favorable changes in selected measures of ventilatory load. These findings describe the integrated physiologic response to the combined strategy and warrant evaluation in prospective controlled studies.

## 1. Introduction

Acute respiratory distress syndrome (ARDS) is characterized by severe impairment of gas exchange and remains associated with substantial morbidity and mortality [1]. Lung-protective ventilation with low tidal volumes and limitation of airway pressures remains the cornerstone of management, with the primary goal of mitigating ventilator-induced lung injury (VILI) [2]. In patients with moderate-to-severe ARDS, early and prolonged prone positioning improves oxygenation and reduces mortality and is therefore recommended as part of standard care [3,4,5]. However, further reductions in ventilatory intensity may be difficult to achieve in selected patients in whom severe gas-exchange impairment is accompanied by refractory hypercapnia and clinically relevant acidemia [6,7].

Although prone positioning improves oxygenation and promotes more homogeneous distribution of ventilation, its effect on carbon dioxide elimination is variable [8]. Importantly, prone positioning is not intended to dissociate carbon dioxide clearance from mechanical ventilation [9]. Therefore, in some patients persistent hypercapnia with acidemia may continue to constrain further reductions in ventilatory intensity [10].

Extracorporeal carbon dioxide removal (ECCO_2_R) has been proposed as a strategy to enable ultra-protective ventilation by reducing ventilatory demand, thereby decreasing driving pressure and mechanical power [11,12]. However, randomized clinical trials of ECCO_2_R in ARDS have not demonstrated improved survival [13,14]. Extracorporeal multiorgan support using the ADVOS system differs conceptually from conventional ECCO_2_R by combining fluid-based carbon dioxide removal with correction of acid–base disturbances, controlled ultrafiltration, and removal of water-soluble and protein-bound toxins [15,16,17]. These additional functions may be particularly relevant in critically ill patients in whom severe respiratory failure coexists with metabolic derangements, acute kidney injury, and fluid overload.

Whether extracorporeal multiorgan support can be safely combined with prone positioning, and what short-term physiologic evolution occurs during such a combined strategy has not been systematically examined. Specifically, it remains unclear whether early correction of hypercapnia and acidosis during prone ventilation may attenuate ventilatory stress and improve physiologic tolerance.

To explore this concept, we conducted a retrospective physiologic study in patients with severe ARDS and multiorgan dysfunction undergoing prone positioning during extracorporeal multiorgan support with the ADVOS system. The primary objective was to characterize the short-term physiologic response to the combined strategy from baseline to completion of the first prone session, focusing on arterial pH, PaCO_2_, oxygenation, driving pressure, and respiratory system compliance. Secondary analyses characterized subsequent physiologic trajectories, hemodynamic and fluid-balance changes, and the technical feasibility of maintaining extracorporeal support during prone positioning. The study was designed to describe the integrated physiologic response to the combined intervention rather than to determine the independent efficacy of either prone positioning or extracorporeal support.

## 2. Materials and Methods

### 2.1. Study Design and Setting

This retrospective, single-center, observational physiologic proof-of-concept study was conducted in the 14-bed medical intensive care unit (ICU) of a tertiary academic hospital between April 2024 and October 2025.

Adult patients with ARDS who underwent prone positioning in combination with extracorporeal multiorgan support were screened for eligibility through electronic ICU records. ARDS management adhered to international guideline recommendations, including lung-protective ventilation and early prone positioning for patients with persistent moderate-to-severe hypoxemia [4,5]. Patients were followed until ICU discharge or death.

The study was approved on the 12 September 2024 by the institutional review board of the Technical University of Munich, Germany (approval number: 178/20S). Given the retrospective nature of the study, the requirement for written informed consent was waived. The study was conducted in accordance with the Declaration of Helsinki and its subsequent amendments.

### 2.2. Patients

Patients were eligible if they were 18 years of age or older, fulfilled Berlin definition criteria for moderate-to-severe ARDS [18], required invasive mechanical ventilation, underwent at least two prone positioning sessions with a target duration of at least 16 h each, and received extracorporeal multiorgan support during prone positioning. The requirement for at least two prone-positioning sessions was intended to allow longitudinal characterization of the combined strategy across two consecutive prone phases through t40. Patients treated concomitantly with veno-venous extracorporeal membrane oxygenation (ECMO), those in whom life-sustaining therapy was withdrawn before completion of the first prone session, or those with missing physiologic data at predefined time points were excluded.

Extracorporeal multiorgan support was initiated according to routine clinical practice based on indications determined by the treating physicians, including hypercapnic acidosis, metabolic instability, and/or fluid overload.

### 2.3. Standard ARDS Management and Prone Positioning Protocol

All patients were managed according to institutional ARDS protocols. Lung-protective ventilation was applied with tidal volumes of 4–6 mL/kg predicted body weight (PBW). Ventilator settings were individualized according to gas exchange, respiratory mechanics, and clinical requirements. Consequently, observed tidal volumes at individual time points could exceed the nominal target range. No study-driven ventilator adjustments were performed. Plateau pressures were limited in accordance with institutional standards and positive end-expiratory pressure (PEEP) was adjusted using the ARDSNet PEEP table [19]. Sedation and neuromuscular blockade were administered as clinically indicated.

Prone positioning was initiated in patients with persistent hypoxemia (PaO_2_/FiO_2_ < 150 mmHg) despite optimization of ventilatory settings. Each prone session targeted a duration of at least 16 consecutive hours. Decisions regarding continuation or repetition of prone positioning were made by the treating team on the basis of oxygenation and overall clinical stability [5]. Ventilator settings were not protocolized for study purposes and remained at the discretion of the treating physicians throughout the observation period.

### 2.4. Extracorporeal Multiorgan Support

Extracorporeal multiorgan support was initiated at the discretion of the treating intensivist based on the overall clinical condition. Indications included a combination of clinically relevant hypercapnic acidosis despite lung-protective ventilation, metabolic/acid–base derangements, acute kidney injury requiring extracorporeal support, and/or fluid overload. It was delivered using the ADVanced Organ Support (ADVOS) system (ADVITOS GmbH, Munich, Germany), an extracorporeal multiorgan support platform comprising interconnected blood, dialysate, and regeneration circuits [20]. ADVOS provides extracorporeal solute and fluid exchange using an albumin-containing dialysate circuit. Carbon dioxide handling occurs through fluid-phase removal of bicarbonate-related species rather than through a membrane lung. In addition, adjustment of dialysate composition permits modulation of acid–base balance, while the system provides renal replacement functions, controlled ultrafiltration, and removal of water-soluble and protein-bound substances [17,21].

Blood flow during treatment ranged between 100 and 300 mL/min. Dialysate composition (i.e., type of alkaline concentrate with varying bicarbonate content) and pH were adjusted according to clinical requirements to facilitate carbon dioxide removal and acid–base correction. Ultrafiltration targets were defined by the treating physicians on the basis of hemodynamic stability and fluid balance goals. Anticoagulation was performed using citrate, as previously described [17]. Operational parameters were documented in the electronic medical record.

### 2.5. Study Timeline and Data Collection

Physiologic variables were recorded at predefined time points relative to initiation of the combined treatment strategy. Baseline (t0) was defined as the last available physiologic assessment obtained in the supine position before initiation of prone positioning and extracorporeal multiorgan support. Prone positioning and ADVOS were initiated clinically as a combined strategy; because both procedures were started within a short operational interval, their exact temporal sequence could not be reliably determined retrospectively. Subsequent study time points (t1, t4, t8, t16, t24, t25, t28, t32, and t40) were defined relative to initiation of the combined treatment strategy. The first prone phase extended to t16, corresponding to completion of the first 16-h prone session. A subsequent supine interval was defined as t24, followed by a second prone phase extending to t40.

The primary physiologic evaluation time point was defined a priori as t16, representing completion of the first prone session under extracorporeal support. All time points were specified before data extraction, and no post hoc redefinition was performed.

Clinical, ventilatory, hemodynamic, and laboratory data were extracted from electronic medical records and ventilator documentation systems. Arterial blood gas analyses were performed as part of routine clinical care.

### 2.6. Physiologic Variables and Definitions

Acid–base assessment included arterial pH, PaCO_2_, bicarbonate concentration, and base excess to characterize both respiratory and metabolic components of acid–base correction during extracorporeal support. Gas exchange variables additionally included PaO_2_/FiO_2_ ratio.

Mechanical ventilation was managed according to lung-protective principles using pressure-controlled mode. Ventilatory parameters included tidal volume (V_T_ expressed as mL/kg PBW), respiratory rate (RR), PEEP, and plateau pressure. Driving pressure (DP) was calculated as the difference between plateau pressure and PEEP:

Driving pressure = P_plat_ − PEEP (1)

Static respiratory system compliance was calculated as

C_rs_ = V_T_/(P_plat_ − PEEP) (2)

Mechanical power was expressed in joules per minute according to the reported formula for pressure-controlled ventilation [22], and was analyzed as a secondary physiologic variable.

MP_PCV_ = 0.098 · RR · V_T_ · (DP + PEEP) (3)

Vasopressor dose was used to monitor hemodynamic trajectory. Fluid management variables included ultrafiltration rate (mL/h) and net 24-h fluid balance. Fluid administration and ultrafiltration targets were determined by the treating physicians and were not protocolized for study purposes. Ultrafiltration rate represented fluid removal through the extracorporeal system, whereas net fluid balance reflected total fluid inputs and outputs over 24 h.

### 2.7. Outcomes

The primary objective was to characterize the short-term integrated physiologic response to the combined strategy from baseline (t0) to completion of the first prone phase (t16). The primary physiologic variables were arterial pH, PaCO_2_, PaO_2_/FiO_2_, driving pressure, and static respiratory system compliance.

Secondary objectives were to characterize the subsequent physiologic trajectory through t40, including acid–base, ventilatory, hemodynamic, and fluid-balance variables, and to assess the technical feasibility of maintaining extracorporeal support during prone positioning. ICU outcomes were reported descriptively.

Technical feasibility and safety were assessed retrospectively from clinical and device documentation. Safety variables extracted retrospectively included interruption of extracorporeal therapy during prone positioning, circuit dysfunction or clotting, vascular-access complications, clinically relevant bleeding, and other documented device-related adverse events.

Exploratory analyses included the definition of a physiologic responder phenotype and its association with ICU survival. These analyses are reported in the Supplementary Materials.

### 2.8. Bias, Study Size, and Statistical Analysis

To minimize selection bias, consecutive eligible patients were included. Time points were predefined before data extraction, and ventilatory management followed standard-of-care protocols without study-driven modifications. All primary variables consisted of objective physiologic measurements routinely documented in clinical practice.

Given the exploratory nature and small sample size, analyses were primarily descriptive and hypothesis-generating. No formal sample size calculation was performed. Missing data were handled using a complete-case analysis approach, and no imputation procedures were applied.

Continuous variables are presented as median and interquartile range (IQR). Normality was assessed using the Shapiro–Wilk test. Paired comparisons between baseline and follow-up time points were performed using the Wilcoxon signed-rank test. Categorical variables were analyzed using Fisher’s exact test. For exploratory survival analyses, odds ratios with 95% confidence intervals were calculated. A two-sided *p*-value < 0.05 was considered statistically significant. No adjustment for multiple comparisons was performed, and all analyses were considered hypothesis-generating. Therefore, *p*-values beyond the prespecified primary physiologic comparisons should be interpreted descriptively and not as confirmatory evidence. Statistical analyses were conducted using IBM SPSS Statistics for Windows, version 28.0.

## 3. Results

### 3.1. Patient Characteristics

**Figure 1:**
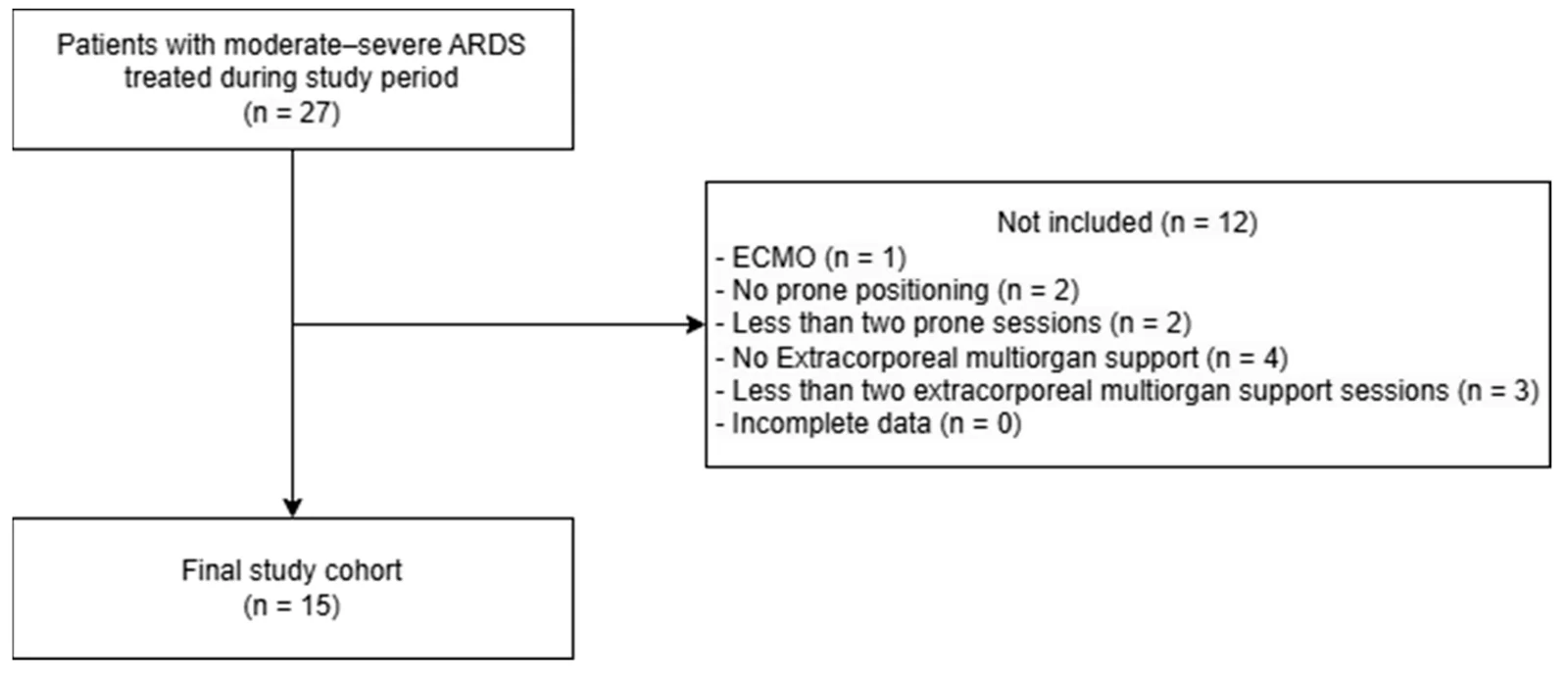
Flow of patients through the study.

During the study period, 27 patients were screened for eligibility. Twelve patients did not meet the complete eligibility criteria because they received concomitant VV-ECMO (n = 1), no prone positioning (n = 2), less than two prone sessions (n = 2), no extracorporeal multiorgan support (n = 4), or less than two extracorporeal multiorgan support sessions (n = 3). Fifteen patients therefore fulfilled the complete eligibility criteria and comprised the final longitudinal cohort (Figure 1).

The cohort represented a highly selected population with substantial respiratory and multiorgan dysfunction. All patients had fulfilled criteria for severe ARDS earlier in their clinical course; however, t0 represented the study baseline rather than the time of initial ARDS severity classification. At t0, 13 of 15 patients had a PaO_2_/FiO_2_ ratio ≤ 100 mmHg, whereas 2 patients had PaO_2_/FiO_2_ ratios of 104 and 114 mmHg, respectively. All patients were invasively ventilated with PEEP ≥ 8 cmH_2_O at t0; median PEEP was 12 cmH_2_O (IQR 10–13). Median baseline PaO_2_/FiO_2_ was 82 mmHg (IQR 74–98), while median PaCO_2_ was 68 mmHg (IQR 59–75) and arterial pH was 7.22 (IQR 7.13–7.28). All 15 cases were microbiologically confirmed as bacterial pneumonia according to routine institutional diagnostic protocols; no patient had viral-, aspiration-, mixed-, or COVID-19-associated pneumonia.

Baseline ventilatory and physiologic parameters at t0 are summarized in Table 1.

The median age was 56 years (IQR 49–61), and 80% were male. At baseline, the median SOFA score was 14 (IQR 12–16). The median interval from initiation of invasive mechanical ventilation to initiation of the combined treatment strategy (extracorporeal multiorgan support and prone positioning) was 16 h (IQR 10–24). The median interval between t0 and initiation of the combined treatment was 3.0 h (IQR 2.0–5.5), reflecting the time required for clinical and operational preparation.

All patients had sepsis and acute kidney injury (AKI), were mechanically ventilated, and required vasopressor support.

### 3.2. Treatment Exposure

All patients underwent at least two prone positioning sessions of 16 h’ duration during extracorporeal multiorgan support. Each patient received a median of three proning sessions (IQR 3–3).

All 15 patients had multiple concurrent indications for extracorporeal multiorgan support, including hypercapnic acidosis (15/15), acute kidney injury with renal replacement requirements (15/15), metabolic acid–base disturbances (15/15), and fluid overload in all the patients (15/15). These indications were multifactorial and overlapping, and no single primary indication was prospectively ranked.

Extracorporeal multiorgan support was maintained continuously for 24 h per session including the prone phase in all patients. Median blood flow during treatment was 300 mL/min (IQR 200–300), and median concentrate flow was 320 mL/min (IQR 320–320). A median dialysate pH of 8.5 (IQR 8.1–8.7) was used. Median ultrafiltration rate during the first prone phase was 100 mL/h (IQR 0–150), and extracorporeal support was continued during both prone and supine intervals without interruption. Each patient received a median of 4 extracorporeal multiorgan support sessions (IQR 3–5).

### 3.3. Integrated Physiologic Response During the Observation Period

#### 3.3.1. Gas Exchange and Acid–Base Status

At baseline (t0), patients exhibited marked hypercapnia and acidemia, with median arterial pH of 7.22 (IQR 7.13–7.28) and PaCO_2_ of 68.1 mmHg (IQR 59.2–74.8). At completion of the first prone session (t16) during extracorporeal multiorgan support, arterial pH increased to 7.41 (IQR 7.37–7.45), corresponding to a median within-patient change of +0.23 (IQR 0.12–0.26; *p* = 0.001). PaCO_2_ decreased to 42.6 mmHg (IQR 37.5–47.9), with a median reduction of −21.0 mmHg (IQR −30.5 to −13.4; *p* = 0.001).

The correction of acidemia occurred in parallel with changes in the metabolic component of acid–base balance. Median bicarbonate increased from 23.4 mmol/L (IQR 19.7–30.2) at baseline to 31.1 mmol/L (IQR 26.5–32.4) at t16, although the paired change did not reach statistical significance (median change +2.1 mmol/L [IQR −1.2 to 9.2]; *p* = 0.065). In contrast base excess increased from −6.0 mmol/L (IQR −9.8 to 3.1) to +4.4 mmol/L (IQR 1.7–7.6), corresponding to a median within-patient increase of +6.3 mmol/L (IQR 2.0–13.0; *p* = 0.013). These concurrent changes indicate that normalization of arterial pH occurred alongside both a reduction in PaCO_2_ and metabolic correction during extracorporeal support. Detailed longitudinal acid–base variables are provided in Supplementary Table S2.

**Figure 2:**
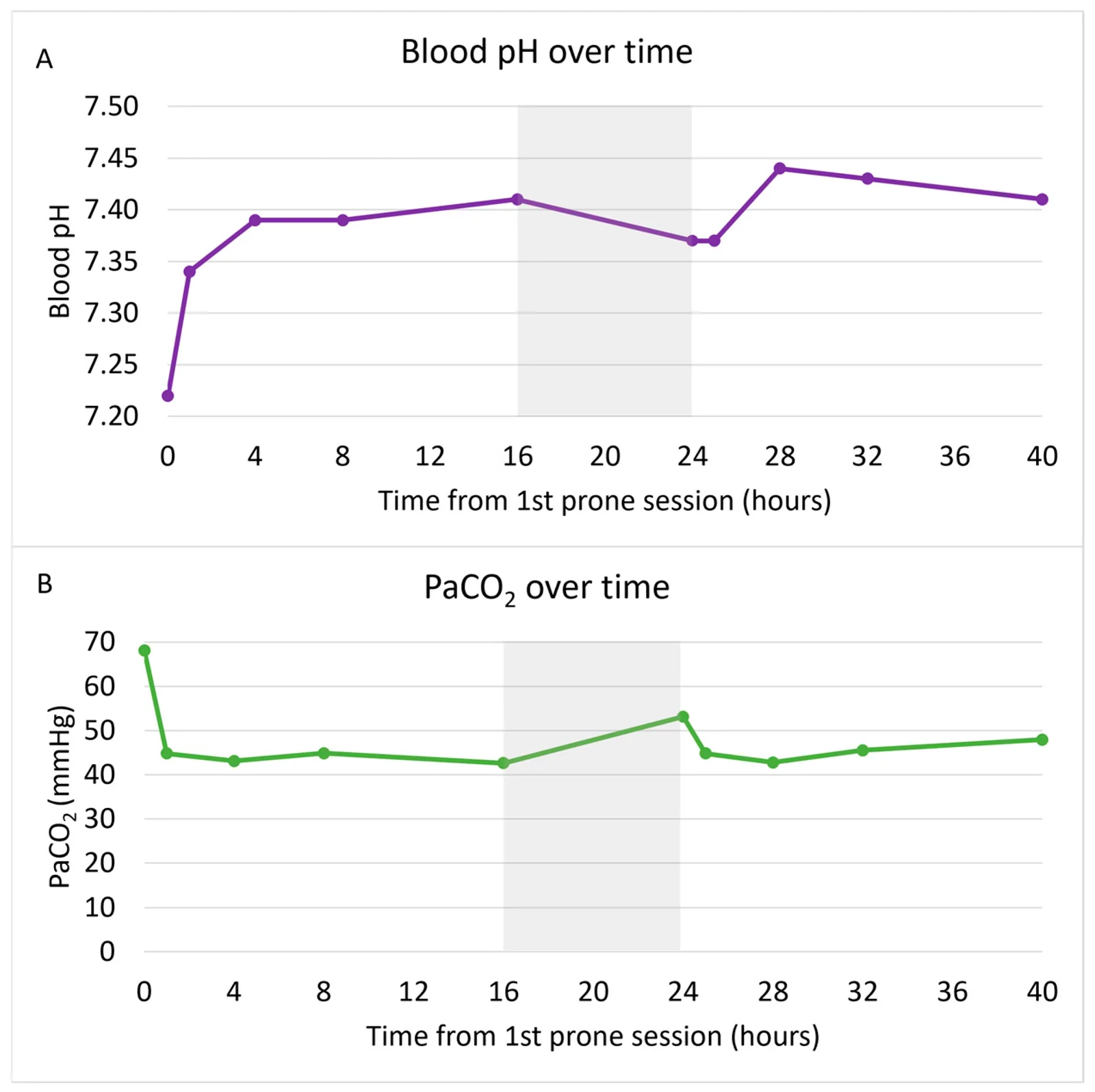
Gas exchange evolution during prone positioning with extracorporeal multiorgan support. Median values are shown. (**A**): Blood pH; (**B**): PaCO_2_. Grey shaded area refers to supine position periods.

The improvements in acid–base status were sustained over time. At t40, arterial pH remained significantly higher than baseline (median 7.41 [IQR 7.37–7.46]; *p* = 0.001 vs. t0), and PaCO_2_ remained lower compared with baseline (median change −20.2 mmHg [IQR −27.1 to −13.0]; *p* = 0.001) (Figure 2).

**Figure 3:**
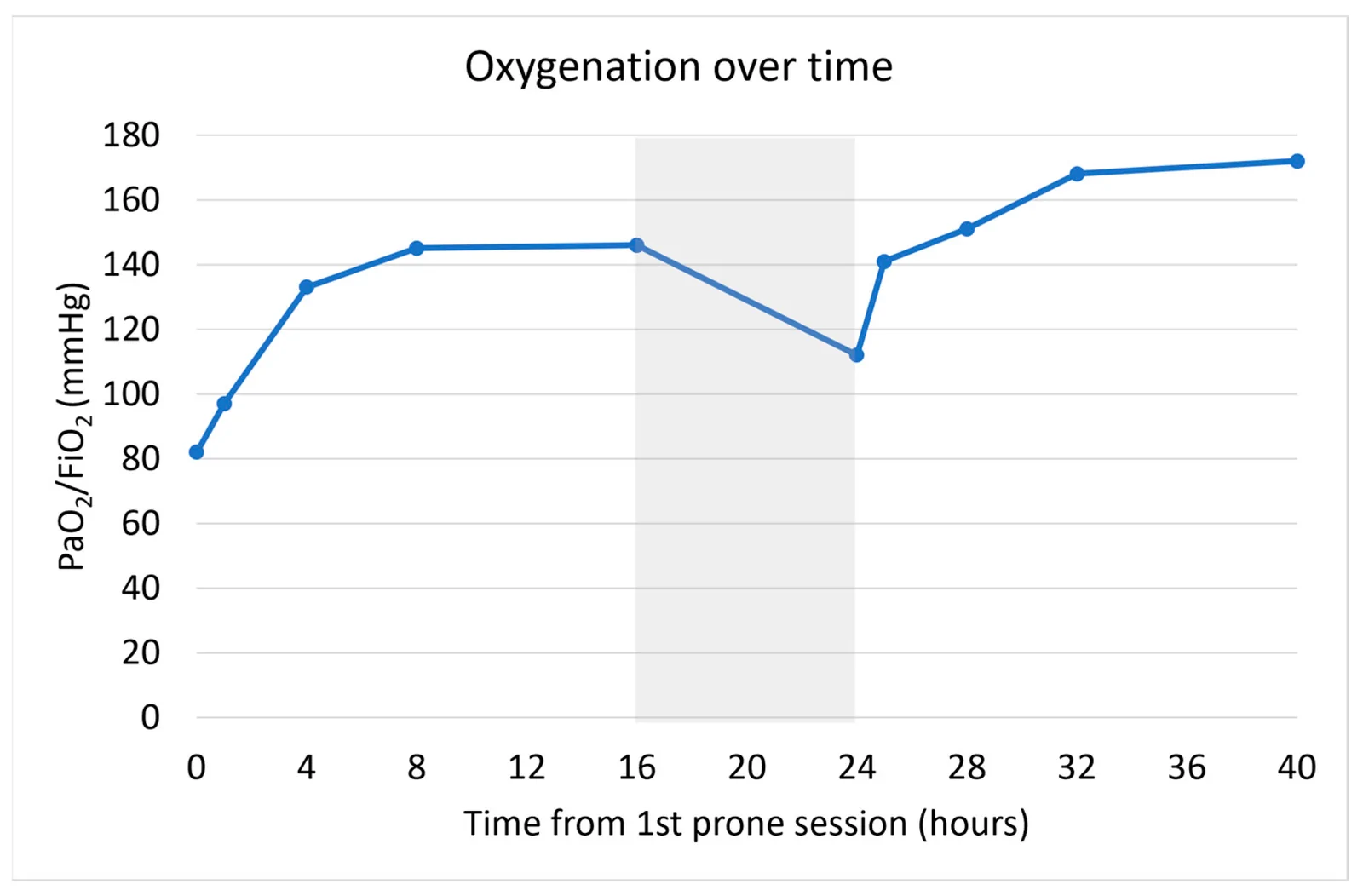
Changes in oxygenation during prone positioning with extracorporeal multiorgan support. Median values are shown. Grey shaded area refers to supine position periods.

Oxygenation improved during the first prone phase. The PaO_2_/FiO_2_ ratio increased from 82 (IQR 74–98) at baseline to 146 (IQR 122–186) at t16 (*p* = 0.001). Values transiently decreased during the supine interval (t24) and subsequently improved during the second prone phase. At t40, the PaO_2_/FiO_2_ ratio reached 172 (IQR 135–214; *p* = 0.001 vs. t0) (Figure 3).

Details are shown in Table 2. Intermediate values across all time points are presented in Table S1.

#### 3.3.2. Ventilatory Variables

**Figure 4:**
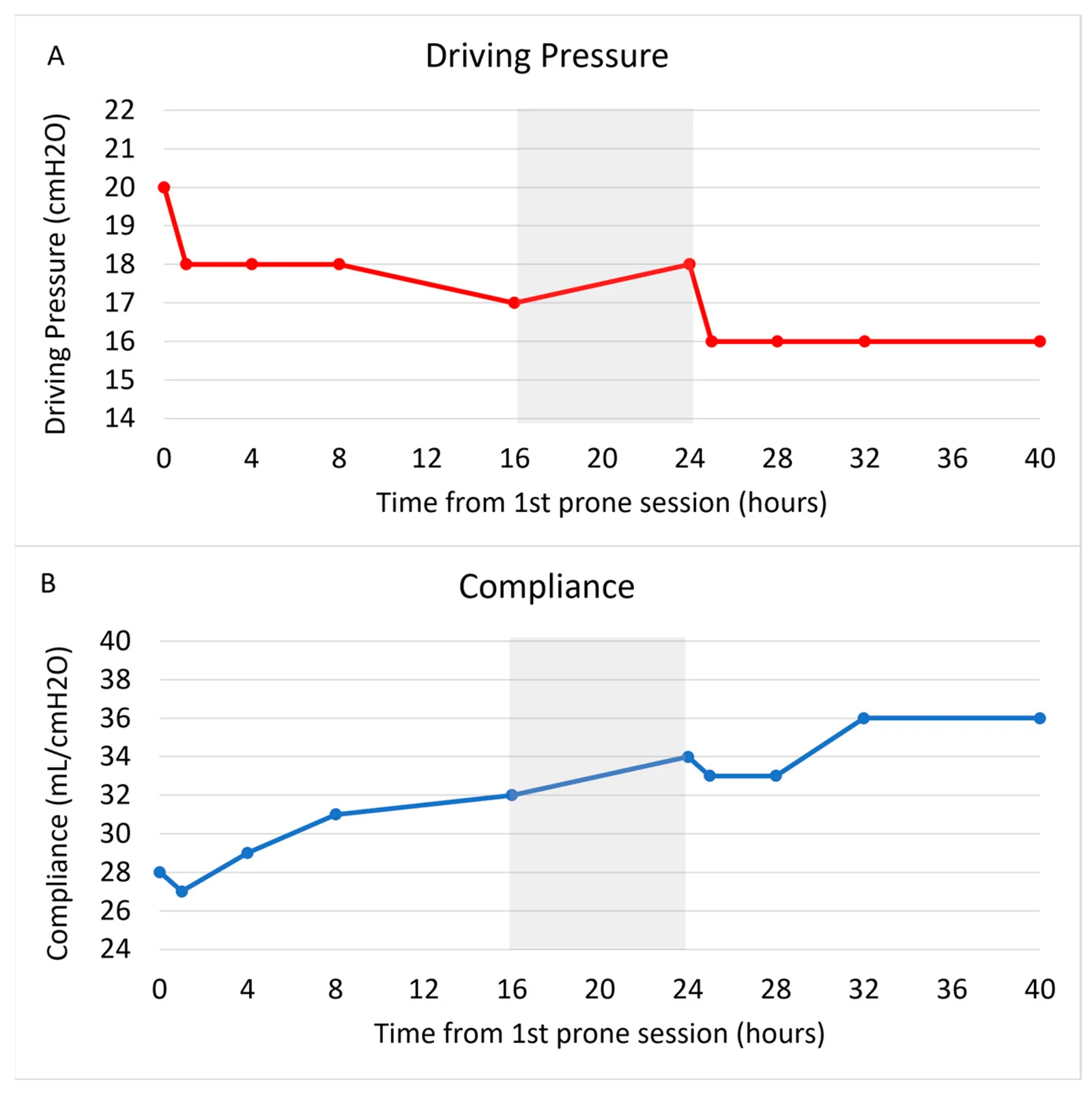
Evolution of ventilatory mechanics during prone positioning under extracorporeal multiorgan support. (**A**): Driving pressure; (**B**): compliance. Median values are shown. Grey shaded area refers to supine position periods.

Driving pressure decreased from 20 cmH_2_O (IQR 18–22) at baseline to 17 cmH_2_O (IQR 15–18) at t16, corresponding to a median change of −2 cmH_2_O (IQR −7 to −2; *p* = 0.003), and remained below baseline at t40 (16 cmH_2_O [IQR 13–18]; *p* = 0.005). Static respiratory system compliance increased from 28 mL/cmH_2_O (IQR 21–39) to 32 mL/cmH_2_O (IQR 29–41) at t16, although the paired change did not reach statistical significance (*p* = 0.090); at t40, compliance was 36 mL/cmH_2_O (IQR 27–44; *p* = 0.041 versus baseline) (Figure 4).

Respiratory rate decreased from 24 breaths/min (IQR 22–24) to 22 breaths/min (IQR 20–22) at t16 (*p* = 0.067) and was lower than baseline at t40 (21 breaths/min [IQR 19–22]; *p* = 0.013). PEEP remained unchanged throughout the observation period at a median of 12 cmH_2_O. Tidal volume did not change significantly.

Mechanical power decreased from 28.5 J/min (IQR 25.5–37.3) at baseline to 25.9 J/min (IQR 23.6–29.6) at t16, corresponding to a median within-patient change of −3.1 J/min (IQR −7.2 to 1.0; *p* = 0.033). At t40, mechanical power was 27.4 J/min (IQR 23.6–30.8), and the difference from baseline was not statistically significant (median change −1.6 J/min [IQR −7.6 to 2.0]; *p* = 0.201).

Further details across all time points are provided in Table S2.

#### 3.3.3. Hemodynamics and Fluid Management

Median vasopressor dose decreased from 0.200 to 0.130 µg/kg/min, corresponding to a median reduction of −0.050 µg/kg/min (IQR −0.395 to −0.020; *p* = 0.016) at t16. A sustained decrease in vasopressor requirements was observed at t40 compared with baseline (median change −0.090 µg/kg/min; *p* = 0.001).

Ultrafiltration was delivered according to individual clinical requirements. Despite extracorporeal fluid removal, median net 24-h fluid balance remained positive during the observation period with 1818 mL/day (IQR −427 to 3949) at t24 and also 1818 mL/day (IQR −410 to 2293) at t40. Forty percent of patients (n = 6/15) achieved a negative fluid balance by the end of the observation period. Data on fluid administration before initiation of prone positioning and extracorporeal support were not available for analysis, which limits further interpretation. Because net fluid balance reflects all fluid inputs and outputs rather than extracorporeal ultrafiltration alone, these trajectories are reported descriptively. Detailed ultrafiltration and fluid-balance data are provided in the Supplementary Information.

#### 3.3.4. Course of Organ Failure Markers

Serum creatinine decreased over the first treatment period (median reduction −36.4%, *p* = 0.014). A decrease in bilirubin was observed (median reduction −11%, *p* = 0.379). Median platelet count decreased from 102/nL (IQR 47–225) to 85/nL (IQR 28–201). These and other laboratory values are summarized in the Supplementary Information (Table S3).

#### 3.3.5. Technical Feasibility and Safety

Extracorporeal multiorgan support was maintained during prone positioning in all 15 patients. No treatment interruption related to prone positioning, catheter dislodgement, or vascular-access malfunction was documented. Review of the available clinical and device documentation identified no documented circuit clotting, premature circuit changes, clinically relevant bleeding, citrate-related complications, electrolyte disturbances, hemodynamic intolerance, or technical alarms leading to treatment termination.

#### 3.3.6. Outcome

Patients were mechanically ventilated for a median of 18 days (IQR 15–29). The median ICU length of stay was 27 days (IQR 16–42) and an ICU survival rate of 53% (8/15 patients) was documented. Clinical outcomes are reported descriptively because the study was not designed or powered to evaluate treatment efficacy.

### 3.4. Exploratory Responder Analysis

According to the exploratory physiologic response criteria (Supplementary Information), 11 patients were classified as physiologic responders and 4 as non-responders.

ICU survival was 64% (7/11) among responders and 25% (1/4) among non-responders. The odds ratio for survival in responders compared with non-responders was 5.25 (95% CI 0.40–69) but this association did not achieve statistical significance in this small exploratory cohort (Fisher’s exact test, *p* = 0.205) and is reported for hypothesis generation only.

Individual patient-level data supporting responder classification are provided in Supplementary Table S5. Differences between groups are presented descriptively in Table S6. Integrated physiologic changes, extended laboratory parameters, and fluid balance data for responders and non-responders are further detailed in Tables S7–S9.

## 4. Discussion

In this retrospective physiologic study of patients with severe ARDS and multiorgan dysfunction, prone positioning combined with extracorporeal multiorgan support was associated with rapid changes across several physiologic domains. Oxygenation improved during prone positioning, with a partial reversal during the intervening supine period and a further increase during the second prone phase. Hypercapnia and acidemia improved rapidly during the first combined treatment period; importantly, normalization of arterial pH occurred alongside both a marked reduction in PaCO_2_ and changes in the metabolic component of acid–base balance. Driving pressure decreased during the first prone period, whereas respiratory rate and respiratory system compliance showed numerical changes that did not reach statistical significance at t16. Mechanical power decreased at t16, although this difference was not sustained at t40. Vasopressor requirements also decreased during the observation period. Finally, extracorporeal support was maintained throughout prone positioning without interruption. These findings characterize the integrated physiologic response to the combined strategy and should not be interpreted as demonstrating the independent effect of either prone positioning or extracorporeal support.

Prone positioning is a well-established intervention in ARDS that improves oxygenation primarily through redistribution of ventilation, reduction in dorsal lung compression, and more homogeneous ventilation–perfusion matching [23]. Moreover, prone positioning combined with standard medical therapy has demonstrated a survival benefit [3], as reflected in national and international guidelines recommending its application for patients with moderate-to-severe ARDS (defined as PaO_2_/FiO_2_ < 150 mmHg and PEEP ≥ 5 cmH_2_O) initiated early after intubation for 16 consecutive hours or more [4,5]. The observed increase in PaO_2_/FiO_2_ ratio in our cohort is consistent with these known effects and falls within the range reported in previous studies of prone positioning in moderate-to-severe ARDS. At baseline, our cohort had a median PaO_2_/FiO_2_ of 82, was ventilated with a median PEEP of 12 cmH_2_O. The combined treatment strategy was initiated at a median of 16 h after intubation, and prone sessions were maintained for 16 h, consistent with current indications for early prolonged prone positioning in moderate-to-severe ARDS. All patients were managed under a lung-protective ventilation strategy.

Yet, protective ventilation may be constrained in selected patients by hypercapnia and clinically relevant acidemia, particularly when respiratory failure coexists with metabolic and hemodynamic instability, and fluid overload [24,25]. Severe hypercapnia may increase pulmonary vascular resistance and right ventricular afterload [26,27,28]. In our cohort, PaCO_2_ decreased rapidly during the combined treatment period, while arterial pH normalized. Our results show that the median time to achieve a pH of 7.35 was 4 h (Supplementary Information), consistent with our previous findings [16]. However, the change in pH cannot be interpreted as a surrogate of extracorporeal CO_2_ removal alone. Base excess increased substantially during the first treatment period, while bicarbonate also increased, indicating a concurrent metabolic contribution to acid–base correction. This is consistent with the mechanism of ADVOS, in which CO_2_ handling occurs through blood–dialysate exchange of bicarbonate-related species while dialysate composition and pH can simultaneously modify systemic acid–base balance. Direct extracorporeal CO_2_ removal (VCO_2_) was not measured in this study; therefore, the relative contributions of extracorporeal CO_2_ removal, metabolic correction, prone positioning, and changes in native pulmonary CO_2_ elimination cannot be quantified. In this context, comparisons with conventional ECCO_2_R trials should be interpreted cautiously because ADVOS does not use a membrane lung and simultaneously modifies acid–base balance and other metabolic domains. In the REST trial, median tidal volumes were comparable; however, a median pH equal to or greater than 7.35 was not achieved until day 5 in the ECCO_2_R group and day 3 in the ventilation-only group, while mean PaCO_2_ remained above 45 mmHg at all reported time points. Notably, in that trial, prone positioning was applied in only 5.5% of patients in the ECCO_2_R group and 13.9% in the ventilation-only group [13]. By contrast, in the BICAR-ICU 2 trial, the target pH (>7.30) was achieved at t12 in the bicarbonate group, while median pH ≥ 7.35 was observed at t24 (t48 in the control group); however, patients in that trial were normocapnic at baseline [29]. Together, these differences reinforce the need to interpret the observed pH trajectory in our cohort as the result of combined respiratory and metabolic changes rather than as a direct measure of extracorporeal CO_2_ removal.

Consistent with this concept, improvements in ventilatory mechanics were observed during the study period. The reduction in driving pressure observed is of physiologic interest but should be interpreted cautiously. Driving pressure is influenced by both respiratory system compliance and ventilatory settings. Improvements in compliance are well described during prone positioning and may account for part of the observed effect [30]. Mechanical power also decreased during the first prone period, although this difference was not sustained at t40. Because ventilator settings were not protocolized for study purposes and no prone-only control group was available, the relative contributions of prone positioning, extracorporeal CO_2_ handling, and clinician-directed ventilatory adjustments cannot be determined. In addition, given the exploratory design and absence of adjustment for multiple comparisons, the *p* = 0.033 finding for mechanical power should not be interpreted as confirmatory evidence of a ventilation-sparing effect. Overall, the data indicate favorable changes in selected components of ventilatory load during the combined strategy but do not establish a direct effect of extracorporeal support on ventilatory intensity.

Beyond respiratory physiology, vasopressor requirements decreased over time. Correction of severe acid–base disturbances may favor vascular tone and hemodynamic tolerance [31]; however, the observational study design precludes causal inference. Similarly, although controlled ultrafiltration was delivered during treatment, median net fluid balance remained positive and fluid administration before initiation of the combined strategy was unavailable. The present data therefore do not support attributing improvements in respiratory mechanics to net fluid removal.

The multiorgan context of this cohort is nevertheless relevant. Severe ARDS frequently occurs in the context of systemic critical illness and multiorgan dysfunction rather than as an isolated pulmonary process [32]. In the present cohort, all patients had sepsis and acute kidney injury and required vasopressor support, illustrating that severe respiratory failure frequently coexists with metabolic, renal, and hemodynamic dysfunction. Extracorporeal support systems designed to provide multiorgan metabolic stabilization may therefore offer an integrated approach that simultaneously addresses respiratory, metabolic, and fluid-related derangements [33]. ADVOS differs from conventional membrane-lung ECCO_2_R devices in that CO_2_ handling occurs within a broader platform providing acid–base modulation, renal replacement functions, controlled ultrafiltration, and removal of water-soluble and protein-bound toxins [20]. Although these systemic effects were not the primary focus of the present investigation, they are concordant with previous results [15,16,17,31]. Whether addressing several physiologic domains simultaneously provides an advantage over more targeted extracorporeal strategies cannot be determined from the present study and warrants prospective evaluation.

The findings should be interpreted within the highly selected phenotype represented by this cohort. Patients had severe pneumonia-associated ARDS with marked baseline hypoxemia and hypercapnic acidemia, together with sepsis, acute kidney injury, and vasopressor dependence. In such patients, strategies that combine established lung-protective interventions with extracorporeal multiorgan support may offer a means of addressing multiple physiologic constraints simultaneously [34,35,36]. But these observations should not be extrapolated to unselected patients with ARDS or to patients in whom isolated hypoxemia can be adequately managed with conventional lung-protective ventilation and prone positioning alone.

Importantly, the approach described here should not be interpreted as an alternative to established ARDS therapies. Lung-protective ventilation and prone positioning remain the cornerstones of ARDS management [2,3,4]. Rather, extracorporeal multiorgan support may represent a potential adjunctive strategy warranting further investigation in selected patients in whom hypercapnic acidemia, metabolic derangements, renal dysfunction, or fluid-management requirements coexists with severe respiratory failure. Future studies are required to determine whether integrated extracorporeal support approaches can improve physiologic tolerance of lung-protective ventilation in broader ARDS populations.

Several limitations merit consideration. First, the absence of a control group treated with prone positioning alone precludes separation of the individual contributions of prone positioning and extracorporeal support. As a result, causal inference regarding the effect of the combined strategy cannot be established. Second, the sample size was small, single-center and was a highly selected retrospective cohort. In particular, eligibility required completion of at least two prone-positioning sessions under extracorporeal support to permit characterization through t40. This requirement may have preferentially selected patients who survived and remained sufficiently stable to continue the combined strategy, introducing selection and survivor bias and limiting generalizability. Third, ventilatory management and extracorporeal treatment settings were determined clinically rather protocolized for study purposes, and may have influenced physiologic trajectories. Fourth, direct extracorporeal VCO_2_ was not measured, preventing quantification of the specific contribution of extracorporeal CO_2_ removal to the observed PaCO_2_ and acid–base changes. Fifth, safety was assessed retrospectively from routine clinical and device documentation rather than through a prospective predefined surveillance protocol; consequently, absence of documented complications cannot exclude under-ascertainment of adverse events. Finally, the present analysis focused on short-term physiologic responses and was not designed or powered to assess clinical outcomes. Detailed long-term and procedural outcomes beyond those reported were not systematically collected as study variables. The exploratory responder and survival analyses are particularly vulnerable to the small subgroup sizes and cohort-selection process and should be considered hypothesis-generating only, without prognostic inference.

Taken together, these findings show that prone positioning can be technically combined with extracorporeal multiorgan support in a highly selected group of patients with severe ARDS and multiorgan dysfunction. The observed trajectory was characterized by improved oxygenation, reductions in PaCO_2_, combined respiratory and metabolic correction of acidemia, and favorable changes in selected measures of ventilatory load, including an early reduction in mechanical power that was not sustained at t40. Because these effects cannot be separated between prone positioning, extracorporeal support, ventilator adjustments, and concurrent clinical management, the present findings should be considered physiologic and hypothesis-generating. Prospective controlled studies are required to determine whether this integrated approach can facilitate lung-protective ventilation or improve clinically relevant outcomes.

## Figures and Tables

**Table 1:** Baseline Characteristics at the Last Supine Assessment Before Initiation of the Combined Treatment Strategy (t0).

Variable		Overall (n = 15)
**Demographics**		
Age, years	(median, IQR)	56 (49–61)
Male	(%, n)	80% (12/15)
Body mass index, kg/m^2^	(median, IQR)	25.7 (24.1–34.6)
**ARDS Characteristics**		
Severity		
PaO_2_/FiO_2_ ≤ 100 mmHg at t0	(%, n)	87% (13/15)
PaO_2_/FiO_2_ > 100–200 mmHg at t0	(%, n)	13% (2/15)
Etiology		
Pneumonia	(%, n)	100% (15/15)
Bacterial	(%, n)	100% (15/15)
Other etiology	(%, n)	0% (0/15)
PaO_2_/FiO_2_ ratio, mmHg	(median, IQR)	82 (74–98)
Time from intubation to initiation of combined treatment, hours	(median, IQR)	16 (10–24)
**Severity of illness**		
SOFA score	(median, IQR)	14 (12–16)
APACHE II score	(median, IQR)	34 (30–37)
Sepsis	(%, n)	100% (15/15)
Acute Kidney Injury	(%, n)	100% (15/15)
Vasopressor use at baseline	(%, n)	100% (15/15)
**Baseline Blood Gas Analysis**		
Arterial pH	(median, IQR)	7.22 (7.13–7.28)
PaCO_2_, mmHg	(median, IQR)	68 (59–75)
PaO_2_ mmHg	(median, IQR)	66 (60–77)
Base excess, mmol/L	(median, IQR)	−6.0 (−9.8–3.1)
Lactate, mmol/L	(median, IQR)	2.6 (1.5–3.6)
**Baseline Ventilatory Parameters**		
Tidal volume, mL/kg PBW	(median, IQR)	5.8 (4.9–7.5)
Respiratory rate, breaths/min	(median, IQR)	24 (22–24)
PEEP, cmH_2_O	(median, IQR)	12 (10–13)
Driving pressure, cmH_2_O	(median, IQR)	20 (18–22)
Compliance (mL/cmH_2_O)	(median, IQR)	28 (21–39)
Mechanical power, J/min	(median, IQR)	28.5 (25.5–37.3)

**Table 2:** Integrated Physiologic Changes from Baseline to the End of the First and Second Prone Sessions.

Variable	Baseline (t0)	End of 1st Prone Session (t16)	Δ t0 → t16	*p* t0 → t16	End of 2nd Prone Session (t40)	Δ t0 → t40	*p* t0 → t40
pH	7.22 (7.13–7.28)	7.41 (7.37–7.45)	+0.23 (0.12–0.26)	0.001	7.41 (7.37–7.46)	+0.18 (0.13–0.27)	0.001
PaCO_2_ (mmHg)	68.1 (59.2–74.8)	42.6 (37.5–47.9)	−21.0 (−30.5–−13.4)	0.001	47.9 (42.9–52.4)	−20.2 (−27.1–−13.0)	0.001
HCO_3_^−^ (mmol/L)	23.4 (19.7–30.2)	31.1 (26.5–32.4)	2.1 (−1.2– 9.2)	0.065	28.8 (27.5–31.3	4.9 (1.4–9.4)	0.017
Base Excess (mmol/L)	−6.0 (−9.8–3.1)	4.4 (1.7–7.6)	6.3 (2.0–13.0)	0.013	4.4 (1.9–6.2)	8.1 (4.9–12.2)	0.003
PaO_2_/FiO_2_ ratio	82 (74–98)	146 (122–186)	+64 (60–91)	0.001	172 (135–214)	+90 (69–137)	0.001
Driving Pressure (cmH_2_O)	20 (18–22)	17 (15–18)	−2 (−7–−2)	0.003	16 (13–18)	−4 (−8–−2)	0.005
Compliance (mL/cmH_2_O)	28 (21–39)	32 (29–41)	4 (1–12)	0.090	36 (27–44)	5 (−1–16)	0.041
Respiratory rate	24 (22–24)	22 (20–22)	−2 (−3–0)	0.067	21 (19–22)	−2 (−3–−2)	0.013
PEEP (cmH_2_O)	12 (10–13)	12 (11–12)	0(−1–2)	0.636	12 (11–14)	0(−1–2)	0.415
Tidal volume (mL/kg PBW)	5.8 (4.9–7.5)	6.4 (5.7–7.3)	0.7 (−0.5–1.0)	0.293	6.7 (6.0–7.4)	1.0 (−0.6–1.3)	0.410
Mechanical Power (J/min)	28.5 (25.5–37.3)	25.9 (23.6–29.6)	−3.1 (−7.2–1.0)	0.033	27.4 (23.6–30.8)	−1.6 (−7.6–2.0)	0.201
Noradrenaline (µg/kg/min)	0.200 (0.100–0.770)	0.130 (0.045–0.355)	−0.050 (−0.395–−0.020)	0.016	0.100 (0.040–0.155)	−0.090 (−0.350–−0.050)	0.001

## Data Availability

The datasets generated and/or analyzed during the current study are not publicly available due to institutional and data protection restrictions but are available from the corresponding author on reasonable request.

## Supplementary Materials

The following supporting information can be downloaded at https://www.mdpi.com/article/10.3390/life16091578/s1: Supplementary Information_Prone_Position. Table S1. Longitudinal Evolution of Physiologic Variables Across Study Timepoints. Median (IQR). Table S2. Longitudinal Metabolic and Laboratory Evolution Across Timepoints. Table S3. Extended Laboratory and Fluid Balance Parameters Across Timepoints. Median (IQR). Table S4. ADVOS System Settings. Table S5. Individual Patient-Level Assessment of Physiologic Responder Criteria. Table S6. Baseline Characteristics from Responders and non-responders at the Last Supine Assessment Before Initiation of the Combined Treatment Strategy (t0). No statistical comparison has been made due to the small sample size and the difference on the number of patients per group. Table S7. Integrated Physiologic Changes from Baseline to the End of the First and Second Prone Sessions for Responders (n = 11). Table S8. Integrated Physiologic Changes from Baseline to the End of the First and Second Prone Sessions for Non-Responders (n = 4). The number of patients is too small to reach statistical significance. Table S9. Extended Laboratory and Fluid Balance Parameters Across Timepoints for Responders and Non-Responders. Median (IQR). No statistical comparison has been made due to the small sample size and the difference on the number of patients per group.

## Abbreviations

The following abbreviations are used in this manuscript:

ADVOS	Advanced Organ Support System
AKI	Acute Kidney Injury
ARDS	Acute Respiratory Distress Syndrome
ECCO_2_R	Extracorporeal Carbon Dioxide Removal
ECMO	Extracorporeal Membrane Oxygenation
ICU	Intensive Care Unit
IQR	interquartile range
PaCO_2_	Partial Pressure of Arterial Carbon Dioxide
PBW	Predicted Body Weight
PEEP	positive end-expiratory pressure
VILI	ventilator-induced lung injury
