# Supplementary material for: Extracorporeal Multiorgan Support During Prone Positioning in Severe ARDS: A Physiologic Proof-of-Concept Study
Source: Life (Basel). 2026 Sep 21;16(9):1578. doi: 10.3390/life16091578 (PMC13608018; doi:10.3390/life16091578)
Supplement: Supplementary file 1 [file life-16-01578-s001.zip › life-4510536-supplementary.pdf]

Supplementary Information

Table S1. Longitudinal Evolution of Physiologic Variables Across Study Timepoints. Median (IQR).

Variable	t0 (supine)	t1 (prone)	t4 (prone)	t8 (prone)	t16 (prone)	t24 (supine)	t25 (prone)	t28 (prone)	t32 (prone)	t40 (prone)
**pH**	7.22 (7.13–7.28)	7.34 (7.28–7.39)	7.39 (7.36–7.48)	7.39 (7.37–7.43)	7.41 (7.37–7.45)	7.37 (7.32–7.43)	7.37 (7.36–7.44)	7.44 (7.37–7.46)	7.43 (7.40–7.46)	7.41 (7.37–7.46)
**$PaCO_2$ (mmHg)**	68.1 (59.2–74.8)	44.8 (40.4–55.1)	43.1 (41.5–49.8)	44.9 (42.8–47.8)	42.6 (37.5–47.9)	53.1 (49.1–56.3)	44.8 (41.7–55.7)	42.8 (40.7–48.7)	45.5 (39.0–51.8)	47.9 (42.9–52.4)
**PaO_2/FiO_2**	82 (74–98)	97 (82–151)	133 (95–150)	145 (112–173)	146 (122–186)	112 (89–123)	141 (112–185)	151 (136–197)	168 (130–212)	172 (135–214)
**Driving Pressure (cmH_2O)**	20 (18–22)	18 (18–20)	18 (16.5–18)	18 (16–18)	17 (14.5–18)	18 (16–20)	16 (14–19)	16 (15.5–18.5)	16 (14.5–18)	16 (13–18)
**Compliance (mL/cmH_2O)**	28 (20–39)	27 (22–34.5)	29 (25–36)	31 (26.5–36)	32 (28.5–41)	34 (22.5–38.1)	33 (25–39)	33 (25–42)	36 (28–44)	36 (26.5–43.8)
**Respiratory Rate (/min)**	24 (22–24)	22 (22–24)	22 (20.5–24)	22 (20–22)	22 (20–22)	22 (21–22)	22 (19–22)	20 (18–22)	20 (18–22)	21 (19–22)
**PEEP (cmH_2O)**	12 (10–13)	11 (10–12)	12 (10–12)	12 (10–13)	12 (11–12)	12 (12–14)	12 (12–12)	12 (12–12)	12 (12–12)	12 (11–14)
**Tidal Volume (mL/kg PBW)**	5.8 (4.9–7.5)	6.8 (5.7–7.6)	6.6 (5.9–7.6)	6.5 (5.9–7.9)	6.4 (5.7–7.3)	6.3 (5.3–7.7)	6.9 (5.8–7.8)	6.3 (6.0–8.1)	6.7 (5.9–8.2)	6.7 (6.0–7.4)
**Mechanical Power (J/min)**	28.5 (25.5–37.3)	29.8 (27.4–32.8)	30.7 (27.4–35.1)	27.7 (23.4–31.5)	25.9 (23.6–29.6)	30.6 (21.5–34.3)	28.3 (21.2–32.5)	26.7 (23.0–32.3)	26.5 (23.4–32.2)	27.4 (23.6–30.8)

Table S2. Longitudinal Metabolic and Laboratory Evolution Across Timepoints

Variable	t0 (supine)	t1 (prone)	t4 (prone)	t8 (prone)	t16 (prone)	t24 (supine)	t25 (prone)	t28 (prone)	t32 (prone)	t40 (prone)
**HCO_3^- (mmol/L)**	23.4 (19.7–30.2)	28.1 (20.3–30.7)	28.2 (24.1–31.6)	27.0 (25.2–28.8)	31.1 (26.5–32.4)	28.4 (25.9–32.7)	28.0 (26.7–30.9)	29.4 (25.6–31.6)	28.3 (27.1–32.8)	28.8 (27.5–31.3)
**Base Excess (mmol/L)**	-6.0 (-9.8–3.1)	3.1 (-6.2–4.8)	4.4 (-2.0–6.5)	2.0 (0.1–3.9)	4.4 (1.7–7.6)	3.6 (0.4–7.4)	3.0 (0.8–5.0)	5.5 (2.6–7.3)	3.6 (0.9–6.7)	4.4 (1.9–6.2)
**K^+ (mmol/L)**	4.6 (4.0–5.0)	4.0 (3.8–4.5)	3.8 (3.6–4.2)	3.9 (3.8–4.1)	3.7 (3.6–4.1)	3.8 (3.7–4.1)	4.2 (3.9–4.5)	4.1 (3.8–4.3)	4.0 (3.8–4.3)	4.0 (3.8–4.2)
**Na^+ (mmol/L)**	134 (131–143)	137 (136–140)	139 (136–140)	137 (136–140)	138 (136–141)	136 (135–140)	137 (136–140)	138 (136–139)	137 (135–139)	138 (136–140)
**Cl^- (mmol/L)**	101 (98–101)	101 (100–102)	101 (100–102)	101 (100–101)	101 (100–102)	101 (100–102)	101 (100–102)	102 (100–102)	101 (100–101)	101 (100–102)
**Ca^{2+} (mmol/L)**	1.13 (0.99–1.22)	1.19 (1.10–1.22)	1.14 (1.09–1.21)	1.15 (1.12–1.21)	1.14 (1.12–1.19)	1.16 (1.07–1.22)	1.15 (1.13–1.26)	1.14 (1.12–1.26)	1.19 (1.13–1.24)	1.21 (1.16–1.25)
**Anion Gap (mmol/L)**	14.2 12.5–15.9)	13.6 (12.3–15.0)	13.8 (10.6–17.0)	13.9 (11.5–15.6)	12.9 (12.3–14.5)	13.4 (11.1–17.3)	12.2 (10.2–14.9)	12.4 (11.1–15.1)	12.3 (11.2–13.9)	13.4 (12.3–16.0)
**Lactate (mmol/L)**	2.6 (1.5–3.6)	2.5 (1.4–6.0)	2.4 (1.8–7.0)	1.6 (1.3–3.4)	1.4 (1.3–2.8)	1.7 (1.4–2.9)	1.5 (1.4–2.0)	1.6 (1.3–2.3)	1.6 (1.2–2.3)	1.5 (1.0–2.5)

Table S3. Extended Laboratory and Fluid Balance Parameters Across Timepoints. Median (IQR).

Variable	t0	t24	t40
**Ultrafiltration (mL)**	n.a.	1248 (0–2495)	2320 (250–3169)
**Fluid balance (mL)**	n.d.	1818 (-426–3949)	1818 (-410–2293)
**Creatinine (mg/dL)**	1.1 (0.9–1.4)	0.7 (0.55–0.9)	n.d.
**Bilirubin (mg/dL)**	3.7 (0.7–8.8)	2.9 (0.75–9.15)	n.d.
**Platelets (×10⁹/L)**	102 (47–225)	85 (27.5–200.5)	n.d.
**aPTT (s)**	39 (34–51)	40 (36–54)	n.d.
**INR**	1.4 (1.2–1.65)	1.5 (1.2–1.65)	n.d.
**Albumin (g/dL)**	2.8 (2.4–3.15)	2.6 (2.25–2.85)	n.d.
**Hemoglobin (g/dL)**	10.2 (8.35–12.25)	8.7 (8.05–11.2)	n.d.
**Total calcium (mmol/L)**	2.14 (1.99–2.16)	2.44 (2.23–2.65)	n.d.

Table S4. ADVOS System Settings.

Variable	t0 - First session	t24 – Second session
**Blood flow (mL/min)**	300 (200–300)	300 (300–300)
**Concentrate flow (mL/min)**	320 (320–320)	320 (320–320)
**Dialysate pH**	8.5 (8.05–8.5)	8.5 (8.1–8.65)
**ADVOS UF rate (mL/h)**	100 (0–150)	100 (50–150)
**ADVOS base bicarbonate**	20 (10–20)	20 (10–20)

Table S5. Individual Patient-Level Assessment of Physiologic Responder Criteria.

Physiologic response was assessed at t16 (end of the first prone positioning period under ADVOS therapy). A patient was classified as a physiologic responder if at least two of the following criteria were met compared with baseline (t0):

(1) reduction in $PaCO_2$ ≥ 20% (or increase in arterial pH ≥ 0.10),

(2) reduction in driving pressure ≥ 3 cmH_2O,

(3) reduction in vasopressor dose ≥ 20%.

To avoid misclassification due to clinical deterioration, response additionally required the absence of relevant worsening in oxygenation or hemodynamic status, defined as a decrease in PaO_2/FiO_2 ≥ 20% and/or an increase in vasopressor dose ≥ 20% compared with baseline.

This composite definition was designed to capture clinically meaningful improvements in gas exchange, lung stress, and circulatory stability during ADVOS-supported prone ventilation.

**Variable**	**Criteria**	**#1**	**#2**	**#3**	**#4**	**#5**	**#6**	**#7**	**#8**	**#9**	**#10**	**#11**	**#12**	**#13**	**#14**	**#15**	**Median**	**IQR**
$PaCO_2$	Reduction ≥ 20%	-41%	-30%	-53%	-25%	-49%	-25%	-28%	-21%	-42%	-48%	-17%	-13%	-14%	-42%	-26%	-28%	(-42– -23)
pH	Increase ≥ 0.10	0.15	0.24	0.36	0.24	0.01	0.09	0.14	0.23	0.37	0.20	-0.07	0.25	0.08	0.27	0.28	0.23	(0.12–0.26)
Driving pressure	Reduction ≥ 3 cmH_2O	-5	-8	-5	-2	-2	-8	-1	-2	-8	0	-2	0	-8	-4	0	-2	(-7– -2)
Vasopressor dose	Reduction ≥ 20%<br>No increase ≥ 20%	-99%	-34%	11%	29%	-20%	-40%	6%	-67%	-86%	-13%	-55%	-88%	-100%	-57%	-25%	-40%	(-76– -17)
PaO_2/FiO_2	No decrease ≥ 20%	133%	41%	78%	18%	73%	144%	81%	145%	200%	98%	61%	76%	184%	149%	61%	81%	(67–145)
**Group**	R: Responder<br>NR: Non-Responder	R	R	R	NR	R	R	NR	R	R	NR	NR	R	R	R	R		

**Survival**		Yes	Yes	No	No	Yes	No	No	Yes	Yes	Yes	No	No	Yes	Yes	No		

Table S6. Baseline Characteristics from Responders and non-responders at the Last Supine Assessment Before Initiation of the Combined Treatment Strategy (t0). No statistical comparison has been made due to the small sample size and the difference on the number of patients per group.

**Variable**		**Overall (n = 15)**	**Responders (n=11)**	**Non-Responders (n=4)**
**Demographics**				
Age, years	(median, IQR)	56 (49–61)	52 (49–61)	57 (50–61)
Male	(%, n)	80% (12/15)	100% (4/4)	100% (4/4)
Body mass index, kg/m2	(median, IQR)	25.7 (24.1–34.6)	25.7 (24.2–34.6)	26.1 (24.4–29.8)
**ARDS Characteristics**				
ARDS severity				
PaO_2/FiO_2 ≤100 mmHg at t0	(%, n)	87% (13/15)	91% (10/11)	75% (3/4)
PaO_2/FiO_2 >100-200 mmHg at t0	(%, n)	13% (2/15)	9% (1/11)	25% (1/4)
Reason for ARDS				
Pneumonia	(%, n)	100% (15/15)	100% (11/11)	100% (4/4)
Others	(%, n)	0%	0%	0%
PaO_2/FiO_2 ratio, mmHg	(median, IQR)	82 (74–98)	76 (62–83)	98 (98–100)
**Severity of illness**				
SOFA score	(median, IQR)	14 (12–16)	14 (13–16)	14 (11–18)
APACHE II score	(median, IQR)	34 (30–37)	34 (29–35)	35 (32–39)
Sepsis	(%, n)	100% (15/15)	100% (11/11)	100% (4/4)
Acute Kidney Injury	(%, n)	100% (15/15)	100% (11/11)	100% (4/4)
Vasopressor use at baseline	(%, n)	100% (15/15)	100% (11/11)	100% (4/4)
**Baseline Blood Gas Analysis**				
Arterial pH	(median, IQR)	7.22 (7.13–7.28)	7.24 (7.13–7.28)	7.18 (7.15–7.23)
$PaCO_2$, mmHg	(median, IQR)	68 (59–75)	68 (55–77)	68 (65–71)
PaO_2 mmHg	(median, IQR)	66 (60–77)	65 (57–77)	69 (65–75)
Base excess, mmol/L	(median, IQR)	-6.0 (-9.8–3.1)	-6.3 (-10.1–3.1)	-5.6 (-7.0– -0.7)
Lactate, mmol/L	(median, IQR)	2.6 (1.5–3.6)	2.6 (1.6–3.5)	2.4 (1.5–3.4)
**Baseline Ventilatory Parameters**				

Tidal volume, mL/kg PBW	(median, IQR)	5.8 (4.9–7.5)	6.2 (5.4–8.8)	5.1 (4.9–5.5)
Respiratory rate, breaths/min	(median, IQR)	24 (22–24)	24 (22–24)	23 (22–25)
PEEP, cmH_2O	(median, IQR)	12 (10–13)	12 (10–13)	12 (12–12)
Driving pressure, cmH_2O	(median, IQR)	20 (18–22)	22 (18–23)	19 (18–19)
Compliance (mL/cmH_2O)	(median, IQR)	28 (21–39)	31 (21–42)	25 (21–29)
Mechanical power, J/min	(median, IQR)	28.5 (25.5–37.3)	28.5 (26.7–37.3)	28.1 (24.0–33.6)
**Outcomes**				
Mechanical ventilation, days	(median, IQR)	18 (15–29)	24 (17–29)	14 (12–21)
ICU length of stay, days	(median, IQR)	27 (16–42)	28 (17–42)	14 (12–23)
ICU mortality (%, n)	(%, n)	47% (7/15)	36% (4/11)	75% (3/4)

Table S7. Integrated Physiologic Changes from Baseline to the End of the First and Second Prone Sessions for Responders (n=11).

Variable	Baseline (t0)	End of 1st prone session (t16)	Δ t0→ t16	p t0→t16	End of 2nd prone session (t40)	Δ t0→ t40	p t0→t40
**pH**	7.24 (7.13–7.28)	7.42 (7.38–7.48)	+0.24 (0.12–0.28)	**0.004**	7.42 (7.38–7.48)	+0.18 (0.13–0.29)	**0.004**
**$PaCO_2$ (mmHg)**	68.1 (55.1–77.6)	40.3 (37.1–45.3)	-22.8 (-30.5– -12.9)	**0.004**	43.8 (41.6–51.7)	-25.1 (-30.6– -9.6)	**0.045**
**PaO_2/FiO_2 ratio**	76 (62–83)	144 (120–187)	+64 (60–95)	**0.045**	179 (151–214)	+95 (85–138)	**0.004**
**Driving Pressure (cmH_2O)**	22 (18–23)	17 (14–18)	-5 (-8– -2)	**0.009**	15 (12–17)	-6 (-9– -3)	**0.037**
**Compliance (mL/cmH_2O)**	31 (21–42)	31 (29–37)	2 (-2– 8)	0.203	36 (29–44)	5 (-4– 16)	0.824
**Respiratory rate**	24 (22–24)	22 (20–22)	-2 (-2– -3)	**0.030**	21 (19–22)	-2 (-4– -1)	**0.038**
**PEEP (cmH_2O)**	12 (10–13)	12 (12–13)	0 (0–2)	0.151	12 (12–14)	0 (0–3)	0.499
**Tidal Volume (L/Kg PBW)**	6.2 (5.4–8.8)	6.4 (5.7–8.0)	0.1 (-0.8–0.9)	0,756	6.7 (5.9–7.5)	-0.1 (-1.5–1.3)	0.625
**Mechanical Power (J/min)**	28.5 (26.7–37.3)	24.8 (22.6–28.2)	-3.7 (-8.6– -1.2)	**0,018**	26.3 (22.3–28.8)	-6.7 (-9.4– -0.4)	0.068
**Noradrenaline (μg/kg/min)**	0.200 (0.100–0.770)	0.060 (0.030–0.205)	-0.060 (-0.395– -0.040)	**0.029**	0.060 (0.025–0.105)	-0.100 (-0.350– -0.070)	**0.004**

Table S8. Integrated Physiologic Changes from Baseline to the End of the First and Second Prone Sessions for Non-Responders (n=4). The number of patients is too small to reach statistical significance.

Variable	Baseline (t0)	End of 1st prone session (t16)	Δ t0→ t16	p t0→t16	End of 2nd prone session (t40)	Δ t0→ t40	p t0→t40
**pH**	7.18 (7.15–7.23)	7.35 (7.30–7.39)	+0.17 (0.09–0.21)	0.855	7.38 (7.34–7.41)	+0.19 (0.11–0.24)	0.100
**$PaCO_2$ (mmHg)**	68.0 (64.5–70.7)	47.9 (43.4–51.3)	-17.8 (-23.7– -14.4)	0.100	51.4 (49.5–53.5)	-14.8 (-17.4– -13.1)	0.100
**PaO_2/FiO_2 ratio**	98 (98–100)	172 (154–181)	+71 (52–73)	0.100	144 (126–180)	+43 (28–77)	0.100
**Driving Pressure (cmH_2O)**	19 (18–19)	18 (18–18)	-2 (-2– -1)	0.181	19 (18–19)	-1 (-1– 0)	1.000
**Compliance (mL/cmH_2O)**	25 (21–29)	38 (31–43)	12 (10– 13)	0.100	33 (26–47)	8 (5– 17)	0.100
**Respiratory rate**	23 (22–25)	21 (20–23)	-1 (-3– 0)	0.371	21 (20–23)	-2 (-2– -2)	0.100
**PEEP (cmH_2O)**	12 (12–12)	11 (10–12)	-1 (-1–0)	0.371	11 (10–13)	-1 (-2–1)	0.789
**Tidal Volume (L/Kg PBW)**	5.1 (4.9–5.5)	6.5 (5.9–7.1)	0.9 (0.7–1.3)	0.100	6.8 (6.1–7.4)	1.1 (1.0–1.5)	0.100
**Mechanical Power (J/min)**	28.1 (24.0–33.6)	29.6 (28.3–32.0)	-0.3 (-2.0–2.9)	0.855	31.0 (27.3–35.9)	2.4 (1.9–3.3)	0.100
**Noradrenaline (μg/kg/min)**	0.255 (0.158–0.538)	0.310 (0.160–0.463)	-0.005 (-0.165– -0.033)	0.855	0.250 (0.155–0.348)	-0.030 (-0.205– -0.013)	0.855

Table S9. Extended Laboratory and Fluid Balance Parameters Across Timepoints for Responders and Non-Responders. Median (IQR). No statistical comparison has been made due to the small sample size and the difference on the number of patients per group.

	Responders			Non-Responders		
**Variable**	**t0**	**t24**	**t40**	**t0**	**t24**	**t40**
**Ultrafiltration (mL)**	n.a.	3041 (490–3570)	2320 (250–3169)	n.a.	638 (0–1316)	1410 (375–2415)
**Fluid balance (mL)**	n.d.	408 (-962–3518)	-13 (-1286–2172)	n.d.	2657 (1713–3634)	2220 (2035–2501)
**Creatinine (mg/dL)**	1.1 (0.8–1.3)	0.7 (0.5–0.8)	n.d.	1.3 (1.1–1.6)	0.9 (0.8–1.1)	n.d.
**Bilirubin (mg/dL)**	3.7 (0.7–6.8)	2.9 (0.75–5.5)	n.d.	7.1 (2.7–11.3)	7.1 (2.2–11.6)	n.d.
**Platelets (×10^9/L)**	109 (55–225)	85 (37–200.5)	n.d.	52 (2–160)	54 (15–131)	n.d.
**aPTT (s)**	39 (33–51)	40 (36–54)	n.d.	40 (38–47)	42 (40–48)	n.d.
**INR**	1.4 (1.15–1.65)	1.5 (1.2–1.65)	n.d.	1.35 (1.28–1.50)	1.35 (1.18–1.55)	n.d.
**Albumin (g/dL)**	2.9 (2.45–3.3)	2.5 (2.25–2.95)	n.d.	2.6 (2.3–2.9)	2.7 (2.4–2.8)	n.d.
**Hemoglobin (g/dL)**	11 (8.85–12.45)	8.8 (8.25–11.25)	n.d.	8.5 (7.7–9.7)	8.1 (8.0–8.9)	n.d.
**Total calcium (mmol/L)**	2.07 (1.95–2.16)	2.44 (2.26–2.74)	n.d.	2.3 (2.1–2.5)	2.4 (2.2–2.5)	n.d.
